# Robotic Assisted Laparoscopic Donor Nephrectomy: An Update

**DOI:** 10.1007/s11934-025-01263-7

**Published:** 2025-04-05

**Authors:** Armando Salim Munoz Abraham, Mahmoud Morsi, Rodrigo Vianna

**Affiliations:** 1 Department of Surgery, University of Miami Miller School of Medicine, Jackson Memorial Hospital, Miami, FL USA; 2 Miami Transplant Institute, University of Miami Miller School of Medicine, 1801 NW 9th Ave, 7th Floor, Miami, FL 33136 USA; 3 Jackson Memorial Hospital, Miami, Florida USA

**Keywords:** Robotic-assisted nephrectomy, Kidney donor, Kidney transplant, Living donor, Outcomes, Review

## Abstract

**Purpose of the Review:**

To review the outcomes of robotic-assisted laparoscopic donor nephrectomy in the published literature.

**Recent Findings:**

Robotic-assisted laparoscopic donor nephrectomy has demonstrated to be a safe, efficient and effective technique of minimally invasive surgery, that offers multiple advantages to the surgeon, and good outcomes for the kidney donor and recipient. Although still a recent technique, it has been adopted by multiple centers worldwide.

**Summary:**

Robotic donor nephrectomy studies demonstrated consistent perioperative outcomes with low complication rates. Mean operative time was approximately 208 min, which is within acceptable limits. Mean warm ischemia time of 3.84 min remains well below the threshold for graft function preservation. Blood loss during is consistently low, below 150 mL, and conversion to open surgery remains rare, with a rate of 1.08%. These findings suggest that robotic-assisted procedures are feasible and safe for donor nephrectomy.

**Supplementary Information:**

The online version contains supplementary material available at 10.1007/s11934-025-01263-7.

## Introduction

Kidney transplantation remains the gold standard treatment for patients with End-Stage Renal Disease (ESRD), significantly improving survival rates and quality of life compared to dialysis [1, 2]. Over the past few decades, the number of living donor kidney transplants has increased, driven by greater awareness and acceptance of organ donation among family members and close contacts of ESRD patients [3]. Compared to deceased donor kidney transplants, living donor kidney transplantation offers shorter warm ischemia times, improved graft function, higher long-term survival rates, and lower rejection risks [2, 4].

However, donor safety and surgical morbidity remain key concerns in living kidney donation, as healthy individuals undergo major surgery to provide a life-saving organ. The advent of minimally invasive surgical techniques has revolutionized the donor nephrectomy process by reducing recovery times, minimizing postoperative pain, improving cosmetic outcomes, and lowering the risk of complications [5]. Laparoscopic donor nephrectomy (LDN)—introduced in 1995—has long been the gold standard for kidney procurement [6]. Despite its widespread adoption, laparoscopy presents several limitations, including restricted instrument maneuverability, two-dimensional visualization, and ergonomic discomfort for surgeons [5].

The introduction of robotic-assisted surgery, particularly with the da Vinci Surgical System (Intuitive Surgical Inc.), has overcome many of these challenges by integrating three-dimensional visualization, high-definition magnification, and EndoWrist^®^ technology to enhance precision and dexterity during surgery [1, 7]. Since its first adoption in transplant surgery in 2001, robotic-assisted laparoscopic donor nephrectomy (RDN) has gained traction across multiple centers worldwide, demonstrating promising surgical outcomes and improved donor safety [1, 7].

This review provides an updated analysis of the surgical techniques, perioperative outcomes, donor safety, and recipient benefits of RDN as reported in peer-reviewed literature.

## Materials and Methods

A systematic review was conducted to assess the surgical techniques, perioperative outcomes, and safety of RDN. The study followed the Preferred Reporting Items for Systematic Reviews and Meta-Analysis (PRISMA) guidelines [8]. A comprehensive literature search was performed using PubMed (National Library of Medicine), Google Scholar, Scopus, and ISI Web of Knowledge, covering publications from 2019 to 2024. The search was conducted using the following keywords: “Robotic-assisted nephrectomy,” “kidney donor,” “kidney transplant,” “living donor,” “outcomes,” and “review.”

Studies were selected based on specific inclusion and exclusion criteria. Inclusion criteria required original single-center or multicenter studies published in English, focusing on surgical techniques, perioperative outcomes, and donor/recipient safety. Comparative studies between robotic-assisted and laparoscopic or open donor nephrectomy were also included, along with relevant systematic reviews. Exclusion criteria comprised case reports, editorials, letters to the editor, studies with incomplete or insufficient data, and those focusing on pediatric donor nephrectomy.

From the selected studies, key data were extracted and analyzed to evaluate the safety and efficacy of RDN. Extracted data included preoperative donor selection criteria, surgical techniques, sample size, donor demographics (age, gender, kidney laterality), operative metrics (operative time, warm ischemia time (WIT), estimated blood loss (EBL), perioperative and postoperative complications, length of hospital stay (LOS), and recipient outcomes such as delayed graft function (DGF), last estimated glomerular filtration rate (eGFR), and creatinine levels. The final dataset was reviewed to determine the advantages of robotic-assisted donor nephrectomy compared to conventional approaches.

## Results

### Donor Selection and Preoperative Work-Up

Donor candidates were required to be healthy individuals above 18 years of age, with the majority being relatives, spouses, or close friends of the recipients [9, 10]. Before acceptance, all potential donors underwent a comprehensive preoperative evaluation, including physical examination, laboratory tests, psychological assessment, and legal screening to rule out coercion in donation [9, 10].

A computed tomography (CT) angiogram of the abdomen and pelvis was routinely performed to assess renal anatomy, vascular structures, kidney size, presence of multiple vessels, and any lesions or anomalies. Some institutions also incorporated 3D vascular reconstruction for enhanced visualization. Additionally, a Tc-99 m DTPA renal scan was utilized to evaluate glomerular filtration rates (GFR) of both kidneys, ensuring that the dominant kidney (≥ 55% function) was preserved for the donor [9–11].

While the left kidney was the preferred choice for procurement due to more favorable vascular anatomy, in cases where the right kidney demonstrated better structural characteristics or function, it was selected instead. All donor candidates underwent multidisciplinary board review to confirm their eligibility for surgery [9–11].

### Surgical Techniques

RDN technique has evolved across institutions but remains consistent in its core principles. The patient is positioned in lateral decubitus, depending on the kidney selected for donation. The da Vinci Robotic Surgical System (Intuitive Surgical, Sunny Valley, CA) was used in all reported cases [9–18].

**Fig. 1:**
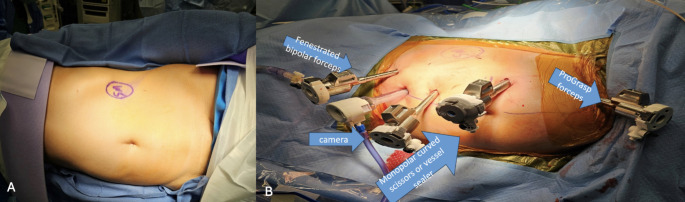
**A**) Patient positioning, **B**) Port placement

Most centers employed three to four robotic trocars: two to three 7–8 mm trocars and one 12 mm trocar for the robotic stapler. Typical port placements included subxiphoid, periumbilical paramedian, iliac fossa, and suprapubic positions. More recent studies reported using a Pfannenstiel incision for kidney graft extraction, offering better cosmetic and postoperative outcomes [9]. Some centers used GelPort-assisted extraction, while others preferred a fascial incision that was preserved until graft retrieval [11] (Fig 1).

### Operative Outcomes

The reviewed studies included a total of 1,846 robotic-assisted donor nephrectomy cases. Most donors were female (59%), with a mean age of 48.9 years (range 18–72 years). The left kidney was procured in 91% of cases due to its anatomical advantages.

Operative times varied between institutions, but warm ischemia time (WIT) remained consistently low, averaging 3.8 min. Estimated blood loss (EBL) was generally less than 150 mL, reflecting the minimally invasive nature of the technique. Conversion to open surgery was rare, reported in 0–3.9% of cases.

### Postoperative Outcomes

Hospital LOS remained short, with most donors being discharged within 3 to 5 days postoperatively. Perioperative complications were minimal, with reported rates ranging from 0 to 17%, the majority classified as Clavien-Dindo Grade 1 or 2, requiring no major interventions.

### Recipient Outcomes

Among kidney transplant recipients, delayed graft function (DGF) was observed in 1.7–11.5% of cases. At last follow-up, mean estimated glomerular filtration rate (eGFR) ranged from 57 to 64 mL/min/1.73 m², and serum creatinine levels were stable between 1.1 and 1.37 mg/dL. Vascular and ureteral complications were rare but reported in a small subset of cases (Tables 1, 2).

**Table 1:** Study characteristics and operative data

Author	Year of Publication	Country	Sample Size	Donor Age (mean)	M/F	Kidney Laterality (Left/Right)	Operative Time (min)	WIT (min)	EBL (ml)	Conversion to Open (%)
Centonze, L. et al. [10]	2023	Italy	154	56	45/109	134/20	210	3.8	20	0
Lecoanet, P. et al. [12]	2022	France	69	49	32/37	N.R.	202.7	6.3		0
Olumba, F. et al. [13]	2023	United States	75	45.3	27/48	67/8	182		< 150	0
Papa, S. et al. [14]	2023	United States	77	44.1	19/58	59/18	301	3.2	60	0
Pelegrin, T. et al. [15]	2022	France	118	49	43/75	116/2	120	4	50	0
Serni, S. et al. [11]	2021	Italy	36	55	16/20	28/8	230	NR	NR	0
Spaggiari, M. et al. [9]	2022	United States	1090	35.7	490/600	1037/37	159	3	50	0.6
Takagi, K. et al. [16]	2021	Netherlands/Japan	103	54	42/61	83/20	180	NR	78	3.9
Windisch, O. et al. [17]	2022	Switzerland	72	51.3	22/50	59/13	287	3.6	NR	0
Zeuschner, P. et al. [18]	2020	Germany	52	54	16/36	41/11	223	3	NR	1.9
Overall Mean (All Studies)	N/A	Multiple	1846	49.34	N/A	91% Left	209.47	3.8	< 150 (estimated)	0.64

M., Male; F., Female; Min, minutes; WIT, warm ischemia time; EBL, estimated blood loss; ml, mililiters; N/A, not applicable; NR, not reported

**Table 2:** Donor postoperative and recipient outcomes

Author	LOS (days)	Transfusion (%)	Follow-up (months)	Donor Complications (%)	Last eGFR (ml/min/1.73)	Last Creatinine (mg/dL)	DGF Rate (%)	Recipient Complications
Centonze, L. et al. [10]	4 (3–5)	0.6	NR	8.4	NR	NR		NR
Lecoanet, P. et al. [12]	6.3	NR	NR	15	NR	NR	12	NR
Olumba, F. et al. [13]	1.8 (0.7)	0	12	5	NR	NR	6	Renal artery stenosis, vascular complications, perirenal hematoma
Papa, S. et al. [14]	2.2	0	12	5.2	64	1.1	NR	NR
Pelegrin, T. et al. [15]	5 (4–5)	0.85	24	7.6	60		NR	NR
Serni, S. et al. [11]	6 (5–7)	0	24	25	57	1.2	2.6	1 graft loss from renal artery thrombosis unrelated to RDN operation
Spaggiari, M. et al. [9]	3	0.4	15	17.3	NR	1.2	2.6	13 cases of rejection, 4 vascular complications, 6 ureteral complications
Takagi, K. et al. [16]	3 (3–4)	NR	NR	0	NR	NR	NR	NR
Windisch, O. et al. [17]	3.8 (1.4)	NR	1	1.3	NR	1.2	NR	NR
Zeuschner, P. et al. [18]	5 (2–12)	NR	NR	5.7	NR	1.1	1.7	5 patients transplant rejection, 3 died (suicide, myocardial infarction, septic shock)
Overall Mean (All Studies)	3.8	0.30	14.6	9.05	60.3	1.16	4.98	Renal artery stenosis, vascular complications, rejection, ureteral complications (rare)

LOS, length of stay; eGFR, estimated glomerular filtration rate; DGF, delayed graft function; NR, not reported

## Discussion

RDN has emerged as a viable alternative to conventional LDN, offering enhanced visualization, improved surgeon ergonomics, and greater precision. The reviewed studies demonstrate the increasing adoption of robotic techniques, highlighting their consistent perioperative outcomes and low complication rates. The mean operative time for RDN across studies was approximately 208 min, slightly longer than LDN, but within acceptable clinical limits. The mean warm ischemia time (WIT) of 3.84 min remains well below the threshold for graft function preservation, indicating that robotic platforms effectively minimize ischemic injury. Blood loss during RDN is consistently low, generally below 150 mL, and conversion to open surgery remains rare, with a reported rate of only 1.08%. These findings suggest that robotic-assisted procedures are technically feasible and safe for donor nephrectomy.

Postoperative recovery following RDN is comparable to laparoscopic techniques, with an average length of hospital stay of 3.8 days. The incidence of perioperative complications was 9.05%, with most classified as minor (Clavien-Dindo Grade 1 or 2), requiring little to no additional intervention. This suggests that RDN does not increase perioperative morbidity and supports its role as a minimally invasive technique. One of the key concerns in donor nephrectomy is its impact on kidney transplant recipients. The analysis shows that delayed graft function (DGF) occurred in 4.98% of recipients, a rate within the expected range for living donor transplants [19]. Furthermore, renal function at last follow-up was well-preserved, with an average estimated glomerular filtration rate (eGFR) of 60.3 mL/min/1.73 m² and stable serum creatinine levels around 1.16 mg/dL. These findings suggest that robotic-assisted nephrectomy does not negatively affect recipient graft outcomes and is a safe surgical option for kidney transplantation.

Despite its clinical advantages, cost remains a significant barrier to the widespread adoption of RDN. The initial capital investment for robotic surgical systems, such as the da Vinci Surgical System, ranges from $1.5 million to $2.5 million, with annual maintenance costs of $100,000–$170,000 and per-case disposable instrument costs of $1,500 to $2,000 [20]. While RDN has been associated with shorter hospital stays and reduced complication rates, the prolonged operative time (on average, 30 min longer than LDN) increases anesthesia and operating room costs, adding to the overall financial burden [20]. High-volume transplant centers may achieve better cost-per-case efficiency, particularly when robots are used across multiple surgical specialties, but the financial feasibility of RDN in low-volume centers remains uncertain. Future strategies such as robotic system leasing, increased competition among manufacturers, and cost-sharing models could enhance affordability and accessibility.

In addition to economic concerns, ethical considerations must be addressed in the implementation of robotic-assisted donor nephrectomy. Living kidney donors are healthy individuals undergoing major surgery for no personal medical benefit, making fully informed consent essential. Donors should be provided with comprehensive information on the risks and benefits of RDN, including the current evidence on safety, efficacy, and surgeon experience. Disparities in robotic surgery access raise additional ethical concerns, as robotic technology is not widely available in all hospitals, creating potential inequities in donor care. Patients at non-robotic centers may feel pressured to travel to high-volume robotic centers, placing additional financial and logistical burdens on them [21].

Moreover, robotic surgery has a steep learning curve, requiring specialized training and experience [22]. The ethical obligation to ensure patient safety necessitates structured training programs and standardized competency assessments before surgeons perform robotic donor nephrectomy independently. Transparent disclosure regarding surgeon experience with robotic procedures is crucial in maintaining patient trust and ethical surgical practices. Finally, the potential for bias toward robotic surgery due to marketing influence and financial incentives must be acknowledged. Decision-making should be guided by objective clinical evidence rather than institutional pressure or surgeon preference.

Overall, RDN has demonstrated excellent safety and efficacy, with low morbidity, rapid recovery, and favorable recipient outcomes. However, challenges related to cost-effectiveness, accessibility, and ethical considerations must be addressed before widespread adoption can be achieved. Future research should focus on long-term donor and recipient outcomes, economic evaluations, and comparative studies to further define the role of robotic surgery in kidney transplantation.

## Conclusion

Robotic-assisted donor nephrectomy has proven to be a safe, efficient, and minimally invasive technique with low perioperative morbidity and excellent recipient outcomes. It offers advantages over laparoscopic techniques in terms of enhanced visualization, superior dexterity, and reduced surgeon fatigue, making it an increasingly preferred choice for donor nephrectomy in specialized centers.

However, challenges remain regarding cost-effectiveness, training requirements, and broader accessibility. Future studies should focus on long-term donor and recipient outcomes, economic analyses, and comparative effectiveness studies to further define the role of robotic surgery in kidney transplantation. Overall, the evidence supports RDN as a highly viable and promising alternative to traditional laparoscopic donor nephrectomy and has already been adopted by many centers around the world as the standard of care.

## Key References

- Spaggiari M, Garcia-Roca R, Tulla KA, Okoye OT, Di Bella C, Oberholzer J, Jeon H, Tzvetanov IG, Benedetti E (2022) Robotic assisted living donor nephrectomies: a safe alternative to laparoscopic technique for kidney transplant donation. Annals of surgery. 2022 Mar 1;275(3):591–5.
-
  - Provides the largest original study demonstrating the advantages of robotic nephrectomy – their outcomes support the use of robotic donor nephrectomy.
  - Highlights very important steps when selecting donors appropriately – provides a guideline for donor selection.
- Di Sandro S, Catellani B, Caracciolo D, Esposito G, Odorizzi R, Olivieri T, Assirati G, Guidetti C, Magistri P, Mori G (2024) The robotic living donor kidney donation: technical aspects and results. European Journal of Transplantation. 2024 Oct 2(2):90–7.
-
  - Describes thoroughly the robotic surgical technique – provides the reader with very specific details on how to perform the procedure.
- South C, Megafu O, Moore C, Williams T, Hobson L, Danner O, Johnson S (2025) Robotic Surgery in Safety-Net Hospitals: Addressing Health Disparities and Improving Access to Care. The American Surgeon™. 2025 Apr;91(4):639–43.
-
  - Highlights a potential barrier for the widespread advancement of Robotic transplantation – May create awareness on finding options to make robotic surgery available for all the population in case the robotic donor nephrectomy becomes standard of care.

## Electronic Supplementary Material

Below is the link to the electronic supplementary material.

Supplementary Material 1

Supplementary Material 2

Supplementary Material 3

## Data Availability

No datasets were generated or analysed during the current study.
