# Supplementary material for: Robotic Assisted Laparoscopic Donor Nephrectomy: An Update
Source: Curr Urol Rep. 2025 Apr 5;26(1):35. doi: 10.1007/s11934-025-01263-7 (PMC11971126; doi:10.1007/s11934-025-01263-7)
Supplement: Supplementary file 1 — Supplementary Material 1 [file 11934_2025_1263_MOESM1_ESM.docx]

| Author | Year of Publication | Country | Sample Size | Donor Age (mean) | M/F | Kidney Laterality (Left/Right) | Operative Time (min) | WIT (min) | EBL (ml) | Conversion to Open (%) |
| --- | --- | --- | --- | --- | --- | --- | --- | --- | --- | --- |
| Centonze, L. et al | 2023 | Italy | 154 | 56 | 45/109 | 134/20 | 210 | 3.8 | 20 | 0 |
| Lecoanet, P. et al | 2022 | France | 69 | 49 | 32/37 | N.R. | 202.7 | 6.3 |  | 0 |
| Olumba, F. et al | 2023 | United States | 75 | 45.3 | 27/48 | 67/8 | 182 |  | <150 | 0 |
| Papa, S. et al | 2023 | United States | 77 | 44.1 | 19/58 | 59/18 | 301 | 3.2 | 60 | 0 |
| Pelegrin, T. et al | 2022 | France | 118 | 49 | 43/75 | 116/2 | 120 | 4 | 50 | 0 |
| Serni, S. et al | 2021 | Italy | 36 | 55 | 16/20 | 28/8 | 230 | NR | NR | 0 |
| Spaggiari, M. et al | 2022 | United States | 1090 | 35.7 | 490/600 | 1037/37 | 159 | 3 | 50 | 0.6 |
| Takagi, K. et al | 2021 | Netherlands/Japan | 103 | 54 | 42/61 | 83/20 | 180 | NR | 78 | 3.9 |
| Windisch, O. et al | 2022 | Switzerland | 72 | 51.3 | 22/50 | 59/13 | 287 | 3.6 | NR | 0 |
| Zeuschner, P. et al | 2020 | Germany | 52 | 54 | 16/36 | 41/11 | 223 | 3 | NR | 1.9 |
| Overall Mean (All Studies) | N/A | Multiple | 1846 | 49.34 | N/A | 91% Left | 209.47 | 3.8 | <150 (estimated) | 0.64 |

Table 1. Study characteristics and operative data

M., Male; F., Female; Min, minutes; WIT, warm ischemia time; EBL, estimated blood loss; ml, mililiters; N/A, not applicable; NR, not reported.
