# Supplementary material for: Robotic Assisted Laparoscopic Donor Nephrectomy: An Update
Source: Curr Urol Rep. 2025 Apr 5;26(1):35. doi: 10.1007/s11934-025-01263-7 (PMC11971126; doi:10.1007/s11934-025-01263-7)
Supplement: Supplementary file 2 — Supplementary Material 2 [file 11934_2025_1263_MOESM2_ESM.docx]

| Author | LOS (days) | Transfusion (%) | Follow-up (months) | Donor Complications (%) | Last eGFR (ml/min/1.73) | Last Creatinine (mg/dL) | DGF Rate (%) | Recipient Complications |
| --- | --- | --- | --- | --- | --- | --- | --- | --- |
| Centonze, L. et al | 4 (3-5) | 0.6 | NR | 8.4 | NR | NR |  | NR |
| Lecoanet, P. et al | 6.3 | NR | NR | 15 | NR | NR | 12 | NR |
| Olumba, F. et al | 1.8 (0.7) | 0 | 12 | 5 | NR | NR | 6 | Renal artery stenosis, vascular complications, perirenal hematoma |
| Papa, S. et al | 2.2 | 0 | 12 | 5.2 | 64 | 1.1 | NR | NR |
| Pelegrin, T. et al | 5 (4-5) | 0.85 | 24 | 7.6 | 60 |  | NR | NR |
| Serni, S. et al | 6 (5-7) | 0 | 24 | 25 | 57 | 1.2 | 2.6 | 1 graft loss from renal artery thrombosis unrelated to RDN operation |
| Spaggiari, M. et al | 3 | 0.4 | 15 | 17.3 | NR | 1.2 | 2.6 | 13 cases of rejection, 4 vascular complications, 6 ureteral complications |
| Takagi, K. et al | 3 (3-4) | NR | NR | 0 | NR | NR | NR | NR |
| Windisch, O. et al | 3.8 (1.4) | NR | 1 | 1.3 | NR | 1.2 | NR | NR |
| Zeuschner, P. et al | 5 (2-12) | NR | NR | 5.7 | NR | 1.1 | 1.7 | 5 patients transplant rejection, 3 died (suicide, myocardial infarction, septic shock) |
| Overall Mean (All Studies) | 3.8 | 0.30 | 14.6 | 9.05 | 60.3 | 1.16 | 4.98 | Renal artery stenosis, vascular complications, rejection, ureteral complications (rare) |

Table 2. Donor postoperative and recipient outcomes

LOS, length of stay; eGFR, estimated glomerular filtration rate; DGF, delayed graft function; NR, not reported.
