# Supplementary figures and images for: Robotic Assisted Laparoscopic Donor Nephrectomy: An Update
Source: Curr Urol Rep. 2025 Apr 5;26(1):35. doi: 10.1007/s11934-025-01263-7 (PMC11971126; doi:10.1007/s11934-025-01263-7)

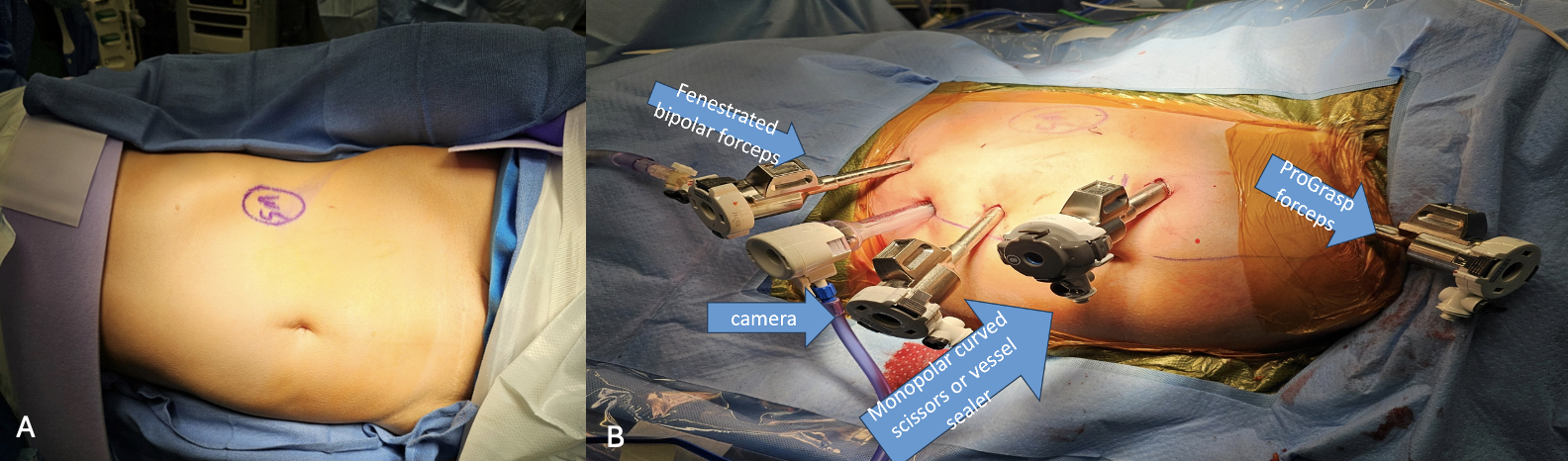

Supplement: Supplementary file 3 — Supplementary Material 3 [file 11934_2025_1263_MOESM3_ESM.png]
